# HAT‐ECG: Hybrid autoencoder‐transformer architecture for ECG arrhythmia classification

**DOI:** 10.14814/phy2.70990

**Published:** 2026-06-23

**Authors:** Shahin Sharbaf Movassaghpour, Masoud Kargar, Ali Bayani

**Affiliations:** ^1^ Department of Computer Engineering, Ta.C. Islamic Azad University Tabriz Iran

**Keywords:** autoencoder, biomedical signal processing, electrocardiogram arrhythmia classification, imbalanced data, patient‐wise evaluation, transformer, wearable monitoring

## Abstract

Accurate and efficient analysis of electrocardiogram (ECG) signals is essential for early detection of cardiac arrhythmias. However, many deep learning models suffer from limited generalization to unseen patients, reduced interpretability, and high computational demands. To address these challenges, we propose HAT‐ECG, a hybrid Autoencoder‐Transformer architecture that integrates unsupervised feature learning with attention‐based temporal modeling. The convolutional autoencoder extracts compact, noise‐robust latent representations of ECG beats, while the Transformer's Multi‐Head Attention mechanism adaptively focuses on diagnostically relevant waveform segments. The model was evaluated on three public datasets: MIT‐BIH, INCART, and the independent PTB Diagnostic ECG Dataset (290 subjects, entirely outside the original datasets). Under standard beat‐wise splitting for benchmarking, HAT‐ECG achieved state‐of‐the‐art accuracies of 99.91% (MIT‐BIH 5‐class), 99.69% (MIT‐BIH AAMI), 99.15% (INCART 3‐class AAMI), and 98.45% (PTB 2‐class). Critically, under strict patient‐wise splitting on MIT‐BIH (completely disjoint training and test patients), the model maintained a realistic accuracy of 90.81% (F1‐score 92.61%), with strong generalization to new patients. With only 0.021 GFLOPs, HAT‐ECG offers an excellent balance of high performance, interpretability, and efficiency, making it highly suitable for real‐time wearable and edge‐device cardiac monitoring. This work advances deep learning for intelligent and deployable arrhythmia classification by combining accuracy, cross‐patient generalization, and computational minimalism.

## Graphical abstract

ECG classification pipeline: Raw signal acquisition, P‑QRS‑T segmentation, feature representation, and heartbeat classification, foundational steps for automated arrhythmia detection.

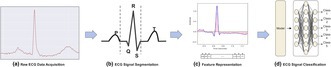

## 1 INTRODUCTION

**FIGURE 1:**
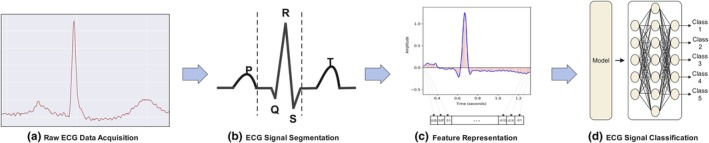
General workflow of ECG signal classification, illustrating common steps in prior studies: (a) acquisition of raw ECG signals, (b) segmentation around the P‐QRS‐T complex, (c) feature representation, and (d) classification into heartbeat classes.

Electrocardiography (ECG) is a fundamental tool for monitoring cardiac activity and detecting arrhythmias, which remain among the leading causes of morbidity and mortality worldwide (Ansari et al., 2023). Accurate and timely arrhythmia detection can significantly reduce the risk of severe cardiac events such as myocardial infarction and sudden cardiac arrest (Lie et al., 2018). Consequently, automated ECG classification systems have gained considerable attention, as they enable real‐time analysis in clinical settings and on wearable devices (Medina‐Avelino et al., 2025). Despite substantial progress in machine learning and deep learning methods, ECG classification remains challenging due to high variability in waveform morphology, severe class imbalance across arrhythmia types, and the need for computationally efficient models that are suitable for resource‐constrained environments (Dhandapani et al., 2025; Essa & Xie, 2021; Xiao et al., 2023). The typical workflow for ECG signal classification involves acquiring raw ECG signals, segmenting the P‐QRS‐T complex, extracting features or vectors for representation, and classifying heartbeats into different categories using machine learning models (Figure 1).

Several challenges hinder the development of robust ECG classification systems. ECG signals are inherently noisy and vary due to patient physiology, electrode placement, and acquisition conditions (Imani et al., 2025; Venton et al., 2021). Heartbeat class distribution is highly imbalanced in widely used datasets such as MIT‐BIH, with normal beats vastly outnumbering pathological ones (Romdhane & Atri Pr, 2020). This imbalance often leads to biased classifiers that perform poorly on rare but clinically important arrhythmias (Ahmad et al., 2021). Most existing methods rely on fixed‐length beat segmentation, which can discard contextual information or introduce artifacts when natural beat lengths vary. Many deep learning models achieve high accuracy but require substantial computational resources, limiting their use in real‐time or embedded scenarios (Chen et al., 2018; Oh et al., 2018).

Over the past few decades, a wide range of methods has been developed for classifying ECG signals and detecting cardiac arrhythmias. Traditional approaches relied on handcrafted feature extraction, where morphological characteristics of the P, QRS, and T waves, along with time‐frequency descriptors, were combined with classical classifiers such as Support Vector Machines (SVM) and k‐Nearest Neighbors (k‐NN) (Jha & Kolekar, 2020; Jung & Lee, 2017; Khalaf et al., 2015). While these methods achieved moderate success, their performance depended on domain‐specific feature engineering and often lacked robustness in noisy or imbalanced scenarios (Jahangir et al., 2025).

With the advent of deep learning, Convolutional Neural Networks (CNNs) became the dominant approach for ECG analysis because they can automatically learn local features from raw signals (Bayani & Kargar, 2024; Huang et al., 2019; Zhang et al., 2022). Several studies have shown that CNN‐based models achieve high accuracy in arrhythmia classification (Atal & Singh, 2020). Similar CNN‐based frameworks have also demonstrated superior performance in other biomedical domains, such as MRI‐based glioma classification (Hoseini et al., 2025). This highlights the general effectiveness of convolutional architectures in medical diagnostics. However, CNNs are limited in capturing long‐term dependencies across cardiac cycles, which are crucial for detecting complex patterns. To address this, Recurrent Neural Networks (RNNs) and variants such as Long Short‐Term Memory (LSTM) networks have been used, showing improvements in temporal modeling (Daduvy et al., 2025; Yildirim et al., 2019). Still, RNN‐based models suffer from slow training and difficulties processing long sequences (Dong & Xie, 2026).

More recently, Transformer architectures have been introduced to ECG analysis, using self‐attention to model long‐range dependencies more effectively (Shah et al., 2025; Zhang, Lin, et al., 2025). Transformers showed superior performance compared to RNNs by providing parallelized computation and better generalization to diverse patient data. Unsupervised representation learning techniques, such as Autoencoders, have also been used to compress ECG signals into high‐level latent features, improving classification efficiency (Hou et al., 2019; Liu et al., 2022).

Despite these advances, several challenges remain. First, the class imbalance problem persists because most public ECG datasets contain far more normal beats than abnormal ones, leading to biased models (Lu et al., 2018; Luo et al., 2021). Second, many studies focus on either feature extraction or classification separately, without integrating both in a unified framework (Rai et al., 2024). Third, computational complexity remains a significant issue, especially in real‐time monitoring applications (Ketu & Mishra, 2024). These limitations motivate the development of hybrid frameworks that combine balanced data handling, feature extraction via Autoencoders, and robust sequence modeling through Transformers, as proposed in this study.

To address these limitations, this paper proposes HAT‐ECG, a Hybrid Autoencoder‐Transformer architecture for ECG arrhythmia classification that integrates efficient representation learning with attention‐based temporal modeling. The key contributions of this work are summarized as follows:

- *Variable‐length beat‐centered segmentation*: A novel segmentation strategy that preserves the natural variability of ECG beats (approximately 300–400 samples around annotated R‐peaks), enhancing signal fidelity and cross‐patient generalization compared to conventional fixed‐length windows.
- *Autoencoder‐based representation learning*: A convolutional autoencoder is utilized to extract compact, noise‐robust, and discriminative features from raw ECG signals, reducing dimensionality while maintaining essential morphological information.
- *Transformer‐based temporal modeling*: A lightweight Transformer module captures both local waveform morphology and long‐range temporal dependencies across beats via multi‐head self‐attention, enabling interpretable and context‐aware classification.
- *AAMI‐compliant evaluation*: The proposed framework is rigorously validated under the AAMI standard, ensuring clinically meaningful arrhythmia grouping and enabling fair, standardized comparison across benchmark datasets.
- *State‐of‐the‐art performance*: HAT‐ECG achieves state‐of‐the‐art results on the MIT‐BIH, INCART, and PTB datasets with minimal computational complexity, demonstrating its suitability for real‐time and wearable cardiac monitoring applications.

## 2 METHODS

**FIGURE 2:**
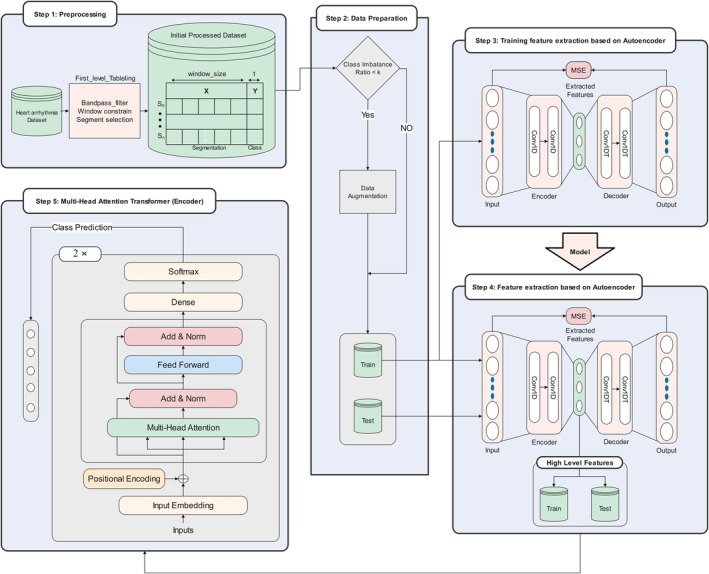
General workflow of the proposed method. The framework includes: (Step 1) signal preprocessing and labeling, (Step 2) data preparation with class imbalance handling and augmentation, (Step 3) unsupervised autoencoder training for beat‐level feature extraction, (Step 4) latent feature extraction for train/test sets, and (Step 5) Transformer‐based classification with positional encoding and multi‐head attention.

The proposed approach aims to improve ECG arrhythmia classification by combining advanced signal processing, representation learning, and deep learning‐based sequence modeling. The overall workflow consists of five main stages: (1) signal preprocessing, (2) data preparation, (3) training a convolutional autoencoder on the training data for feature learning, (4) feature extraction from both training and testing data using the trained encoder, and (5) classification using a multi‐head attention Transformer model (Figure 2).

### 2.1 Datasets

Experiments were conducted on three widely used benchmark datasets for arrhythmia classification: the MIT‐BIH Arrhythmia Database (Moody & Mark, 2001) and the INCART 12‐lead Arrhythmia Database (Goldberger et al., 2000). To further evaluate the generalization capability of the proposed method, the PTB Diagnostic ECG Dataset (Bousseljot et al., 1995; Kachuee et al., 2018) was additionally included under the same experimental protocol. Summary details of these datasets are provided in Table 1.

**TABLE 1:** Summary of the MIT‐BIH, INCART, PTB ECG datasets used in this research.

Dataset	No. of subjects	No. of records	Leads	Sampling rate (Hz)	Classes (AAMI)
MIT‐BIH Moody & Mark (2001)	47	48	2	360	N, S (SVEB), V (VEB), F, Q
INCART Goldberger et al. (2000)	32	75	12	257	N, S (SVEB), V (VEB)
PTB Bousseljot et al. (1995); Kachuee et al. (2018)	290	549	12/15 (3 Frank Leads)	1000	Normal, Abnormal

Evaluation followed the Association for the Advancement of Medical Instrumentation (AAMI) EC57 standard, which provides a consistent framework for arrhythmia classification and performance reporting. According to this standard, individual beat annotations are grouped into five clinically meaningful superclasses: Normal (N), Supraventricular Ectopic Beat (SVEB), Ventricular Ectopic Beat (VEB), Fusion Beat (F), and Unknown Beat (Q) to ensure comparability across studies. Table 2 provides an explicit mapping from the original MIT‐BIH beat annotations to these five AAMI superclasses; the superclass definitions themselves strictly follow the AAMI EC57 standard and are not modified. Raw beat annotations from the MIT‐BIH dataset were mapped to these AAMI superclasses for standardized assessment. Annotated R‐peaks were used for beat‐centered segmentation in both datasets.

**TABLE 2:** Mapping of original MIT‐BIH beat annotations to the five AAMI EC57 superclasses (N, SVEB, VEB, F, Q) used in this study. Individual beat labels are shown only to illustrate how multiple MIT‐BIH annotations are grouped into each AAMI superclass for standardized evaluation.

Original MIT‐BIH beat label	Description (selected)	AAMI class	AAMI standard labels	AAMI beat description
N	Normal beat	Normal	N, L, R, e, j	Normal beat, Left bundle branch block (LBBB), Right bundle branch block (RBBB), Atrial escape, Junctional escape
L	Left bundle branch block			
R	Right bundle branch block			
V	Premature ventricular contraction	VEB	V, E	Premature ventricular contraction (PVC), Ventricular escape
/	Paced beat	Unknown	/, f, Q	Paced beat, Fusion of paced & normal, Unclassifiable beat
‐	‐	SVEB	A, a, J, S	Atrial premature, Aberrated atrial premature, Junctional premature, Supraventricular premature
‐	‐	Fusion	F	Fusion of ventricular & normal beat

*Note*: Blank cells indicate arrhythmias present in AAMI standard but excluded from the 5‐class classification.

#### 2.1.1 MIT‐BIH arrhythmia database

This dataset contains 48 half‐hour, two‐lead ECG recordings from 47 subjects, sampled at 360 Hz with 11‐bit resolution over a 10‐mV range. Each recording was annotated by cardiologists with beat labels and rhythm information, resulting in approximately 110,000 annotated beats. To enable fair comparison with prior studies, two types of evaluations were performed on this dataset: (1) a conventional 5‐class configuration, commonly used in related works, and (2) a 5‐class AAMI standard configuration, in which the original beat annotations were mapped to AAMI superclasses for standardized evaluation (Table 2). In the AAMI configuration, beats were grouped into five superclasses: Normal (N), Supraventricular ectopic beat (SVEB), Ventricular ectopic beat (VEB), Fusion beat (F), and Unknown beat (Q).

#### 2.1.2 INCART database

The INCART dataset comprises 75 annotated 12‐lead ECG recordings, each 30 min in duration and sampled at 257 Hz. Beats are annotated into five AAMI classes: N, SVEB, VEB, F, and Q. In this study, only the N, SVEB, and VEB classes were used for evaluation, as is common in related literature, to ensure fair comparison with other studies. This dataset introduces greater variability in patient signals and arrhythmia patterns, which is valuable for assessing model generalization.

#### 2.1.3 PTB diagnostic ECG dataset

The PTB Diagnostic ECG Dataset is a collection of 549 records from 290 individuals, specifically designed for the diagnosis of cardiovascular abnormalities. Each record within this dataset comprises 15 simultaneously measured signals, including the standard 12 leads. This dataset is primarily categorized into two classes: normal and non‐normal. These classifications help in identifying a range of cardiac conditions. The dataset offers a valuable resource for evaluating models focused on binary classification of ECGs. As detailed in the original documentation, a variety of primary heartbeat categories are present, which are grouped into the two main classes used for evaluation in this study. Table 1 provides a summary of its characteristics, integrated with the other datasets.

### 2.2 Signal preprocessing

ECG signals often contain baseline wander, muscle artifacts, and powerline interference. To mitigate these effects, a 4th‐order Butterworth band‐pass filter (0.5–40 Hz) was applied, which removed low‐frequency drift and high‐frequency noise while preserving P‐QRS‐T morphology. After filtering, signals were normalized to the range [0, 1] using min‐max scaling to stabilize neural network training. The complete preprocessing pipeline is summarized in Table 3.

**TABLE 3:** Preprocessing steps applied to ECG signals.

Step	Method	Parameters	Purpose
Band‐pass filtering	Butterworth	0.5–40 Hz, 4th order	Remove baseline wander and high‐frequency noise
Normalization	Min‐Max	[0,1]	Scale signals for stable neural network training

### 2.3 Data preparation (beat segmentation)

Accurate beat‐level analysis relies on precise identification of the R‐peak, which corresponds to ventricular depolarization. In this study, two segmentation strategies were applied:

#### 2.3.1 Fixed‐length segmentation (300 samples)

In the first setting, each beat was extracted using a constant window size of 300 samples, consisting of 200 samples before and 100 samples after the annotated R‐peak:

- window_size = 200 → number of samples before the R‐peak
- after_size = 100 → number of samples after the R‐peak

These parameters were chosen empirically, as initial experiments demonstrated that this configuration produced the most reliable results. Increasing the window size led to overlap between consecutive beats, while reducing it resulted in partial loss of the P‐QRS‐T morphology. The 200/100 split, therefore, provided a balance between capturing complete beat information and avoiding interference from adjacent beats.

#### 2.3.2 Variable‐length segmentation (300–400 samples)

In the second setting, a variable‐length segmentation was introduced to investigate the impact of beat length variability on model performance. Beat segments were extracted around the R‐peak with a base length of 350 samples and a random padding term $\left(\delta_{i}\sim U\left(-50,50\right)\right)$ applied during training, as defined in Equation (1).

(1) $$x_{i}\left[n\right]=s\left[R_{i}-L_{1}:R_{i}+L_{2}+\delta_{i}\right],\;\delta_{i}\sim U\left(-50,50\right)$$

where $s\left[n\right]$ is the raw ECG signal,$R_{i}$ is the annotated R‐peak,$L_{1}=L_{2}=175$, and $\delta_{i}$ is a random padding. This results in effective beat lengths ranging from 300 to 400 samples, ensuring the full P‐QRS‐T complex is preserved while introducing controlled variability. The rationale for this approach was to evaluate whether the proposed HAT‐ECG model remains robust when faced with natural variations in heart rate and beat morphology.

### 2.4 Handling class imbalance

ECG datasets typically exhibit significant class imbalance, where some arrhythmia categories contain far fewer samples than others. To mitigate this issue and enhance classifier generalization, both sampling and data augmentation strategies were applied.

#### 2.4.1 Sampling strategies

Three resampling approaches were evaluated to balance class distributions:

1. *Oversampling*: Minority class beats are duplicated to match the majority class size.
2. *Undersampling*: The majority class is downsampled to match the smallest class
3. *Hybrid sampling*: A combination of over‐ and undersampling to balance the dataset without excessive duplication.

#### 2.4.2 Data augmentation

To further improve minority class diversity and reduce overfitting, synthetic variations of ECG signals were generated using the following transformations:

- Gaussian Noise Addition: Random Gaussian noise with zero mean and standard deviation of 0.005 was added to each sample, as shown in Equation 2.

(2) $$x_{i}^{\prime }=x_{i}+\varepsilon_{i},\;\varepsilon_{i}\sim N\left(0,\sigma^{2}\right)$$

- Random Scaling: Each signal was multiplied by a random scaling factor α drawn uniformly from the range [0.95, 1.05], as defined in Equation 3.

(3) $$x_{i}^{\prime }=\alpha .x_{i},\;\alpha \sim \cup \left(0.95,1.05\right)$$

- Time Shifting: Signals were shifted along the temporal axis by s samples to simulate phase variations. If s>0, the signal was delayed (shifted right); if s<0, it was advanced (shifted left). Out‐of‐range elements were replaced with zeros, as defined in Equation 4.

(4) $$x_{i}^{\prime }=\left\{\begin{array}{c} x_{i-s},\;\text{if}\;0\leq i-s<n\\ 0,\;\text{otherwise} \end{array}\right.$$

These augmentation techniques preserve the physiological characteristics of ECG waveforms while introducing variability, resulting in a more robust and balanced dataset for training.

### 2.5 Feature extraction using autoencoder

To extract compact and discriminative representations of ECG beats, a convolutional autoencoder was employed. The autoencoder was trained in an unsupervised manner to reconstruct input signals, encouraging the encoder to capture high‐level morphological patterns. These encoded features reduce dimensionality while preserving essential diagnostic information, facilitating efficient and robust classification.

The architecture consisted of stacked convolutional layers in the encoder and mirrored transposed convolutional layers in the decoder, enabling the hierarchical extraction of features from raw ECG segments. Two input configurations were considered based on the beat segmentation strategy:

- *Fixed‐length input* (*300 samples*): In this setting, the encoder compressed the input sequence by a factor of four, resulting in a latent representation of 75 time steps with 64 feature maps. The output dimensionality of the latent layer was therefore 75 × 64.
- *Variable‐length input* (*up to 400 samples*): For longer input sequences, the encoder performed a proportional downsampling, mapping 400 time steps to 100 latent time steps, again with 64 feature maps. The resulting latent representation thus had a dimensionality of 100 × 64.

In both cases, the encoder effectively transformed the input ECG signals into a compact feature space while retaining the P‐QRS‐T morphology. These latent features served as input to the Transformer‐based classifier in the subsequent stage.

### 2.6 Classification using transformer

The encoded features obtained from the convolutional autoencoder were fed into a Transformer‐based classifier. Depending on the beat segmentation, the encoder produced latent representations of size (75, 64) for 300‐sample inputs and (100, 64) for 400‐sample inputs. These feature sequences served as input tokens to the Transformer.

The Transformer employed a multi‐head self‐attention mechanism to capture long‐range dependencies between waveform segments, effectively modeling complex temporal relationships in ECG signals. Since attention mechanisms alone do not retain information about signal order, positional encoding was applied to preserve the temporal structure of the beat sequences.

The classification model was designed with two encoder blocks, each consisting of:

- Multi‐head attention with four attention heads,
- A position‐wise feed‐forward network with 256 hidden units,
- Residual connections and layer normalization to stabilize training

The output sequence from the Transformer encoder was aggregated using global average pooling to generate a compact representation. This representation was then passed through fully connected layers, and the final softmax layer produced class probabilities for each beat in accordance with the AAMI classification standard.

Compared to recurrent architectures such as RNNs or LSTMs, the Transformer provides several advantages:

- Global context modeling: captures dependencies across the entire beat.
- Efficient parallelization enables faster training on large‐scale ECG datasets.
- High generalization ability: improves robustness to inter‐patient variability and beat morphology differences

### 2.7 Train‐test protocol

For Scenarios 1–18, all datasets were first shuffled at the beat level and then split into training and test sets using an 80/20 ratio (beat‐wise evaluation protocol). This setup was used to ensure a fair comparison with prior studies reporting beat‐wise results. To prevent data leakage and ensure a fair generalization assessment, the use of the autoencoder was deliberately divided into two distinct stages. During the pre‐training stage (Step 3), the autoencoder was trained in an unsupervised manner using only the training set, enabling it to learn generic and noise‐robust ECG representations without exposure to test data. In the subsequent feature extraction stage (Step 4), the pre‐trained autoencoder was frozen and used solely as a deterministic encoder to extract high‐level latent features from the training and test sets independently, without any further optimization or parameter updates. This separation ensured that no information from the test set influenced model learning while allowing consistent feature representations to be used for supervised Transformer training and evaluation. A separate instance of the proposed HAT‐ECG framework was trained and evaluated independently on each dataset (MIT‐BIH, INCART, and PTB). Although the network architecture, training procedure, and hyperparameter settings were kept identical across all experiments, model parameters were learned exclusively from the training subset of the corresponding dataset. This protocol was adopted to ensure a fair assessment of the proposed architecture under different ECG acquisition conditions, patient populations, and arrhythmia distributions. Evaluating the same architecture independently across multiple datasets provides a stronger indication of the model's robustness and generalization capability, as consistent performance must be achieved without transferring learned weights or patient information between datasets.

### 2.8 Evaluation metrics

Model performance was evaluated in accordance with AAMI standards using five complementary metrics: Accuracy, Precision, Recall (Sensitivity), F1‐Score, and the area under the Receiver Operating Characteristic curve (ROC‐AUC) (Kargar et al., 2020). Their definitions are provided in Table 4.

**TABLE 4:** Definitions of evaluation metrics used to assess model performance according to AAMI standards, with formulas for Accuracy, Precision, Recall, F1‐Score, and ROC‐AUC.

Metric	Formula
Accuracy	(5) $\text{Accuracy}=\frac{\mathrm{TP}+\mathrm{TN}}{\mathrm{TP}+\mathrm{TN}+\mathrm{FP}+\mathrm{FN}}$
Precision	(6) $\text{Precision}=\frac{\mathrm{TP}}{\mathrm{TP}+\mathrm{FP}}$
Recall	(7) $\text{Recall}=\frac{\mathrm{TP}}{\mathrm{TP}+\mathrm{FN}}$
F1‐Score	(8) $F1\;\text{Score}=\frac{2\times \text{Precision}\times \text{Recall}}{\text{Precision}+\text{Recall}}$
ROC‐AUC	(9) $\mathrm{ROC}-\mathrm{AUC}=\int_{0}^{1}\mathrm{TPR}\left(\mathrm{FPR}\right)d\left(\mathrm{FPR}\right)$

Performance was reported both per AAMI class and using macro‐averaged metrics to ensure equal contribution of each class, regardless of frequency. To reduce patient‐specific bias, the patient‐wise split ensured that subjects were disjoint across training and testing. Additionally, results were averaged across multiple random seeds to ensure the robustness of the reported performance.

## 3 RESULTS

This section presents the experimental design used to validate the proposed HAT‐ECG framework.

### 3.1 Preprocessing and evaluation protocol

All experiments were conducted using the preprocessing and evaluation procedures described in the Methods section. Performance was evaluated under both regular and AAMI‐standard protocols using Accuracy, Precision, Recall, F1‐score, ROC‐AUC, and Cohen's Kappa metrics.

#### 3.1.1 Class distribution before and after sampling

ECG arrhythmia datasets are typically highly imbalanced, with normal beats vastly outnumbering abnormal classes. To address this issue, three resampling strategies were employed: oversampling, undersampling, and hybrid sampling.

**FIGURE 3:**
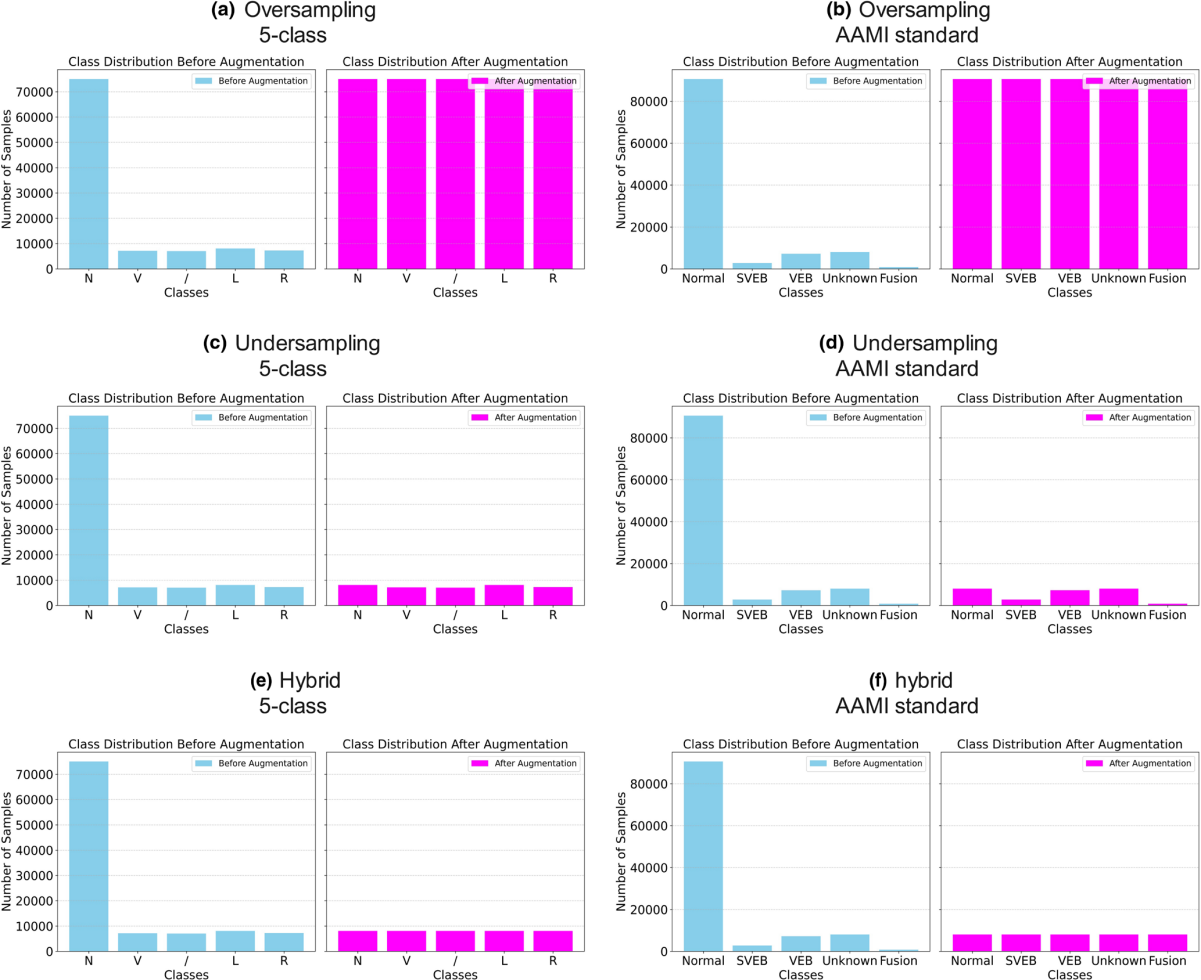
Class distribution of ECG beats before and after applying different sampling strategies under both the 5‐class and AAMI‐standard for MIT‐BIH dataset.

**FIGURE 4:**
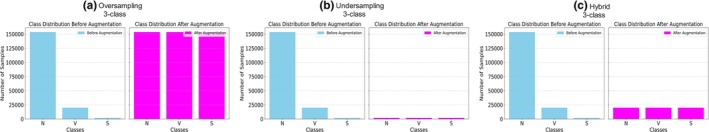
Class distribution of ECG beats before and after applying different sampling strategies for the 3‐class INCART dataset.

In the oversampling strategy, samples from minority classes were duplicated until all classes reached comparable sizes. For the MIT‐BIH dataset, as shown in Figure 3a,b, this approach balanced the data. In the undersampling strategy, samples from the majority class were randomly removed to match the size of the minority classes. The resulting distributions are shown in Figure 3c,d. In the hybrid strategy, a combination of oversampling and undersampling was applied to achieve balance. The resulting distributions are shown in Figure 3e,f. This approach was also applied to the INCART dataset, where Figure 4a–c correspond to oversampling, undersampling, and hybrid strategies, respectively.

### 3.2 Hyperparameters and model configuration

The architectural configurations and training hyperparameters of the autoencoder and Transformer classifier are summarized in Tables 5 and 6. The proposed framework employs a lightweight hybrid architecture designed to balance classification performance and computational efficiency.

**TABLE 5:** Autoencoder architecture and training hyperparameters.

Hyperparameter	Value
Conv1D Layer 1	Filters: 128, kernel_size: 5, activation: Relu, strides: 2, padding: Same
Conv1D Layer 2	Filters: 64, kernel_size: 5, activation: Relu, strides: 2, padding: Same
Dense Encoding Layer	Units: 300, Activation: Relu
Decoder Layers	Conv1DTranspose Layer 1: Filters: 64, kernel_size: 5, activation: Relu, strides: 2, padding: Same
	Conv1DTranspose Layer 2: Filters: 1, kernel_size: 5, activation: Tanh, strides: 2, padding: Same
Compilation	Optimizer: Adam, Loss: MSE
Training	Epochs: 200, Batch_size: 64

**TABLE 6:** Transformer classifier architecture and training hyperparameters.

Hyperparameter	Value
Transformer Blocks	2 blocks
Multi‐Head Attention	num_heads = 4
Feed‐Forward Dim (FFN)	ff_dim = 256
Residual Connections	Enabled
Dense Layer 1	Units: 512, Activation: ReLU
Dense Layer 2	Units: 256, Activation: ReLU
Dense Layer 3	Units: 128, Activation: ReLU
Output Layer	Activation: Softmax
Dropout Rate	0.2
Layer Normalization	epsilon = 1e‐6
Optimizer	Adam
Loss Function	SparseCategoricalCrossentropy
Metrics	Accuracy
Batch Size	32
Epochs	30

To demonstrate computational efficiency, the complexity of each model component was estimated in terms of floating‐point operations per forward pass. As shown in Table 7, the autoencoder requires approximately 0.010 GFLOPs, the Transformer requires 0.011 GFLOPs, and the entire hybrid pipeline requires only 0.021 GFLOPs, confirming its suitability for edge and real‐time applications.

**TABLE 7:** Computational complexity of the proposed hybrid model.

Component	FLOPs (operations)	GFLOPs	Description
Autoencoder	9,606,638	0.010	Feature extraction stage
Transformer	10,864,204	0.011	Classification stage
Total (Hybrid)	20,470,842	0.021	Autoencoder + Transformer combined

### 3.3 Experimental results

**FIGURE 5:**
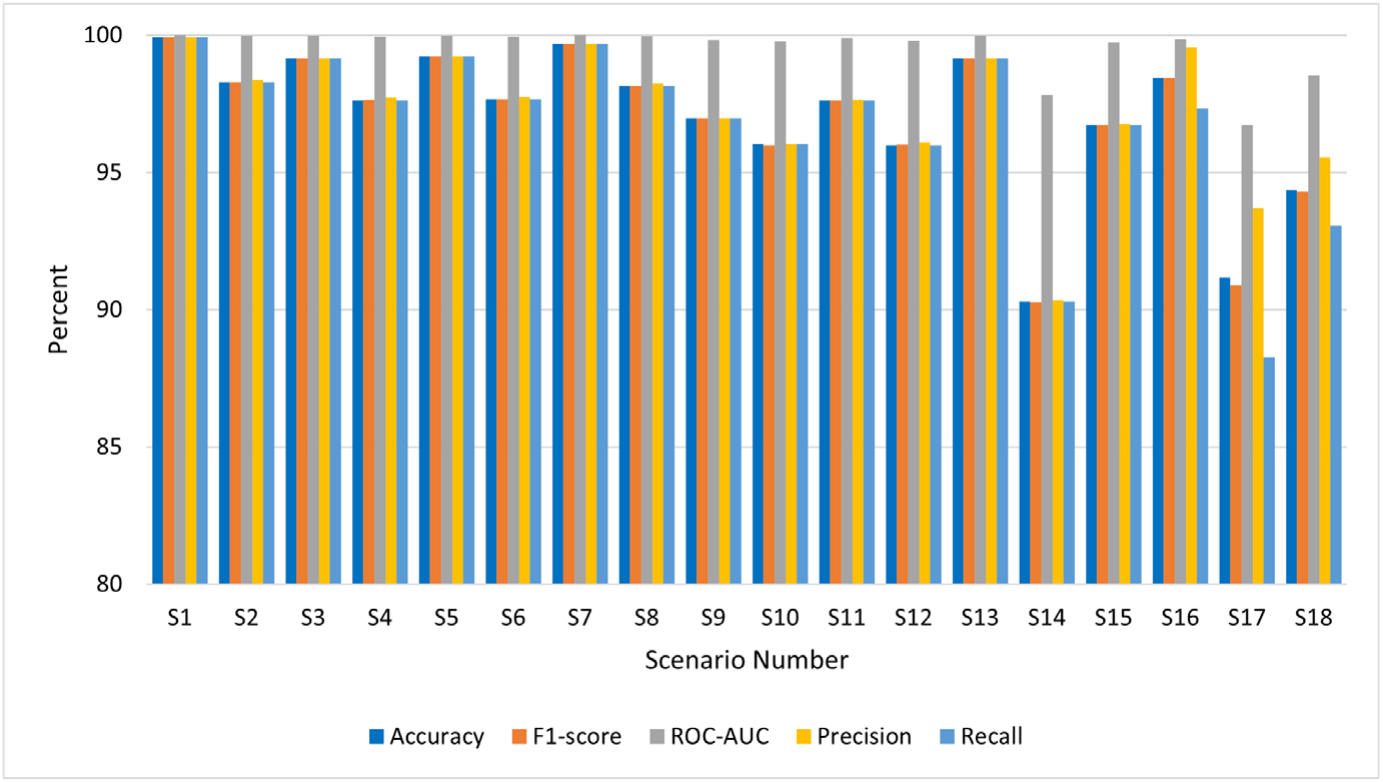
Results of all scenarios from 1 to 18 are compared for the evaluation metrics of Accuracy, F1‐score, ROC‐AUC, Precision, and Recall.

The performance of the proposed model was systematically evaluated across multiple scenarios using the MIT‐BIH, INCART, and PTB datasets. A comparative summary of all scenarios (1–18) and their evaluation metrics, including Accuracy, F1‐score, ROC‐AUC, Precision, and Recall, is presented in Figure 5.

#### 3.3.1 MIT‐BIH database—Regular evaluation (5‐class)

Results under the MIT‐BIH regular beat grouping are summarized in Table 8. Oversampling with fixed‐length 300‐sample segments achieved the best performance, with 99.91% accuracy and an F1‐score of 99.91%, reflecting near‐perfect discrimination. Variable‐length segmentation (300–400 samples) slightly reduced accuracy to 98.28%, but this trade‐off is beneficial for robustness against variability in beat morphology. Both undersampling and hybrid sampling strategies maintained accuracy above 97%, confirming the model's adaptability across various balancing strategies.

**TABLE 8:** Performance on MIT‐BIH under regular evaluation (5‐class).

Sampling method	Segment length	Accuracy	F1‐score	ROC‐AUC	Precision	Recall	Cohen's kappa	Quadratic weighted kappa	Scenario non.
Over	**300**	**99.91**	**99.91**	**100**	**99.91**	**99.91**	**99.89**	**99.91**	**S1**
	300–400	98.28	98.30	99.98	98.39	98.28	97.84	96.83	S2
Under	300	99.15	99.15	99.98	99.15	99.15	98.93	99.06	S3
	300–400	97.62	97.64	99.95	97.74	97.62	97.01	98.12	S4
Hybrid	300	99.23	99.23	99.98	99.23	99.23	99.03	99.26	S5
	300–400	97.66	97.67	99.94	97.77	97.66	97.07	96.78	S6

*Note*: The bolding indicates that these results are particularly noteworthy and represent key findings of our study.

#### 3.3.2 MIT‐BIH database—AAMI Standard evaluation

Performance under the AAMI standard is provided in Table 9. Here, oversampling with 300‐sample fixed segments again yielded the highest accuracy (99.69%) and a Cohen's Kappa of 99.62%, demonstrating excellent alignment with expert labels. Variable‐length segmentation achieved an accuracy of approximately 98%, indicating that compliance with AAMI mappings does not substantially reduce effectiveness. Undersampling resulted in lower scores (approximately 96%), but performance remained strong across all metrics.

**TABLE 9:** Performance on MIT‐BIH under AAMI standard evaluation.

Sampling method	Segment length	Accuracy	F1‐score	ROC‐AUC	Precision	Recall	Cohen's kappa	Quadratic weighted kappa	Scenario non.
Over	**300**	**99.69**	**99.69**	**100**	**99.69**	**99.69**	**99.62**	**99.56**	**S7**
	300–400	98.14	98.16	99.97	98.24	98.14	97.67	98.61	S8
Under	300	96.97	96.97	99.82	96.99	96.97	95.90	94.97	S9
	300–400	96.04	96.0	99.77	96.05	96.04	94.61	95.27	S10
Hybrid	300	97.62	97.63	99.89	97.64	97.62	97.03	97.10	S11
	300–400	95.99	96.01	99.81	96.09	95.99	94.99	93.69	S12

*Note*: The bolding indicates that these results are particularly noteworthy and represent key findings of our study.

#### 3.3.3 INCART database—AAMI Standard evaluation

Table 10 summarizes the results on the INCART dataset. The model achieved the best performance with oversampling (99.15% accuracy), followed by hybrid sampling. Undersampling resulted in a noticeable performance drop, reflecting the loss of information when majority classes are excessively reduced while still maintaining acceptable classification accuracy.

**TABLE 10:** Performance on INCART under AAMI standard evaluation (3‐class).

Sampling method	Accuracy	F1‐score	ROC‐AUC	Precision	Recall	Cohen's kappa	Quadratic weighted kappa	Scenario non.
Over	**99.15**	**99.15**	**99.98**	**99.16**	**99.15**	**98.73**	**98.36**	**S13**
Under	90.31	90.28	97.82	90.36	90.31	85.46	85.22	S14
hybrid	96.73	96.74	99.73	96.78	96.73	95.09	94.63	S15

*Note*: The bolding indicates that these results are particularly noteworthy and represent key findings of our study.

#### 3.3.4 PTB dataset—Regular evaluation (2‐class)

Experiments on the PTB dataset were conducted using a 2‐class (Normal/Abnormal) evaluation to further assess the model's generalization capability on a larger and more diverse dataset. In this study, the PTB dataset was independently partitioned into training and test sets using a beat‐wise splitting strategy, consistent with the experimental protocol applied to the MIT‐BIH and INCART datasets. Results for this evaluation are presented in Table 11. Consistent with findings on the other datasets, oversampling demonstrated the highest performance, achieving 98.45% accuracy and an F1‐score of 98.44%, indicating robust discrimination between normal and abnormal ECG signals. Hybrid sampling also yielded strong results with 94.37% accuracy, while undersampling showed a decrease in performance to 91.17%, reflecting the impact of class balancing strategies on model effectiveness.

**TABLE 11:** Performance on PTB under regular evaluation (2‐class).

Sampling method	Accuracy	F1‐score	ROC‐AUC	Precision	Recall	Scenario non.
Over	**98.45**	**98.44**	**99.85**	**99.56**	**97.33**	**S16**
Under	91.17	90.90	96.72	93.70	88.26	S17
Hybrid	94.37	94.29	98.53	95.55	93.06	S18

*Note*: The bolding indicates that these results are particularly noteworthy and represent key findings of our study.

### 3.4 Patient‐wise evaluation and generalization to unseen subjects

To further evaluate the generalization capability of the proposed HAT‐ECG framework under clinically realistic conditions, an additional patient‐wise evaluation protocol was conducted on the MIT‐BIH dataset. In this setting, subjects were strictly partitioned into mutually exclusive training and testing groups, ensuring that no patient contributed data to both sets. The model was trained exclusively on the training cohort and evaluated only on the held‐out unseen patients, without any form of data sharing between the two groups.

Importantly, while there was no overlap in patient identities between the training and test sets, the distribution of arrhythmia classes was preserved such that all beat types present in the test cohort were also represented in the training cohort. This ensures that evaluation focuses on inter‐subject generalization rather than unseen class discovery, thereby isolating the model's ability to generalize across patient‐specific morphological variability under a consistent set of arrhythmia categories.

Unlike beat‐wise splitting used for benchmarking against prior studies, this protocol enforces inter‐subject independence and prevents the model from learning patient‐specific ECG morphologies such as individual QRS shape variations. This makes the evaluation more representative of real‐world deployment scenarios, where the system encounters completely new individuals.

As summarized in Table 12, the dataset was divided into two disjoint patient groups with no overlap, while maintaining a relatively balanced distribution of arrhythmia classes. Under this strict patient‐wise split, the proposed model achieved an overall classification accuracy of 90.81%, F1‐score 92.61%, Precision 94.11%, and Recall 91.72%, reflecting a more challenging but clinically meaningful evaluation scenario. The results demonstrate that the HAT‐ECG framework retains strong discriminative capability even when applied to unseen patients.

**TABLE 12:** Patient‐wise partitioning of the MIT‐BIH dataset used for evaluating generalization to unseen patients. Training and test sets contain completely disjoint subjects to prevent patient‐level information leakage.

Group	Patient IDs	Description
Training set	101, 106, 109, 112, 114, 115, 116, 118, 119, 122, 124, 201, 203, 205, 207, 208, 215, 220, 222, 223, 230, 232	Patients used for model training
Test set	100, 103, 105, 111, 113, 117, 121, 123, 200, 202, 209, 210, 212, 213, 214, 219, 221, 228, 231, 233, 234	Completely unseen patients used for final evaluation

### 3.5 Dataset statistics

The distribution of heartbeats across MIT‐BIH and INCART datasets is provided in Table 13 (Islam et al., 2024), with mapping to AAMI superclasses. This illustrates the severe imbalance between Normal beats and minority arrhythmia types such as SVEB and Fusion, highlighting the importance of the balancing strategies evaluated.

**TABLE 13:** Distribution of annotated beats across MIT‐BIH and INCART datasets under AAMI mapping.

Classes	Type	Heartbeat name	Heartbeats MIT‐BIH	Heartbeats INCART	Sub‐total MIT‐BIH	Sub‐total INCART
Normal (N)	N	Normal beat	74,658	150,410	90,210	153,676
	L	Left bundle branch block beat	8063	—		
	R	Right bundle branch block beat	7244	3174		
	e	Atrial escape beat	16	—		
	j	Nodal (junctional) escape beat	229	92		
Supraventricular ectopic beat (S)	A	Atrial premature beat	2540	1944	2775	1960
	a	Aberrated atrial premature beat	150	—		
	J	Nodal (junctional) premature beat	83	—		
	S	Supraventricular premature beat	2	16		
Ventricular ectopic beat (V)	V	Premature ventricular contraction	7117	20,013	7223	20,013
	E	Ventricular escape beat	106	—		
Fusion Beat (F)	F	Fusion of ventricular and normal beat	802	219	802	219
Unknown beat (Q)	/	Paced beat	3612	6	3887	12
	f	Fusion of paced and normal beat	260	—		
	Q	Unclassifiable beat	15	6		
Total heartbeats	—		104,897	175,874	—	

### 3.6 Confusion matrices and training trends

**FIGURE 6:**
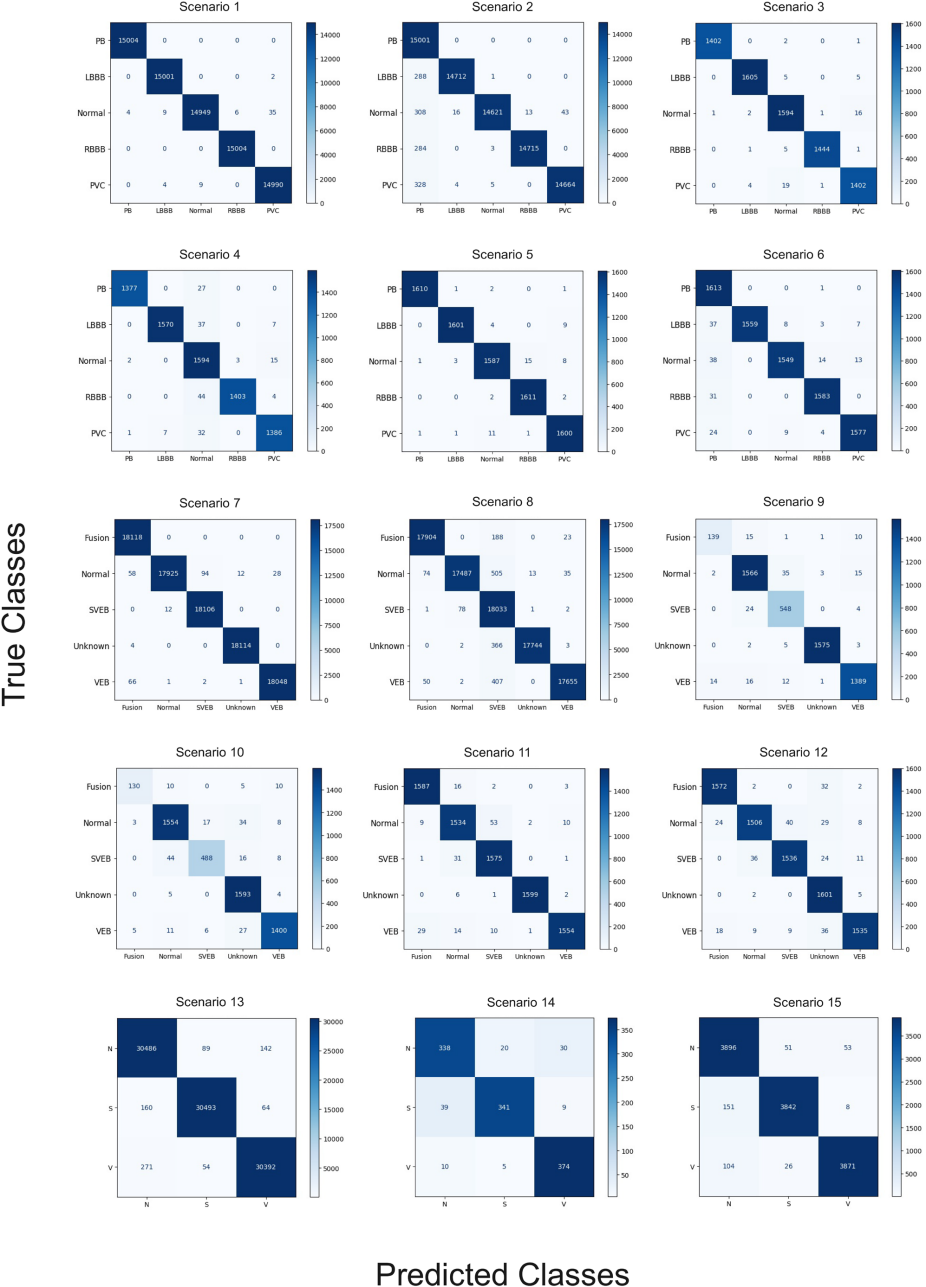
Confusion matrices for all experimental scenarios (1–15), illustrating the classification performance of the proposed HAT‐ECG model under both the 5‐class and AAMI‐standard configurations.

**FIGURE 7:**
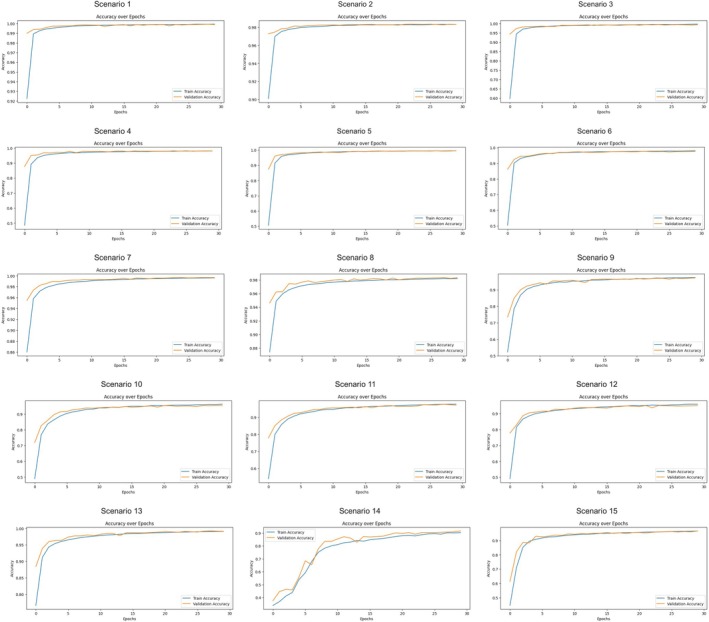
Training and validation accuracy curves across all experimental scenarios (1–15), illustrating model convergence behavior of the proposed HAT‐ECG framework.

**FIGURE 8:**
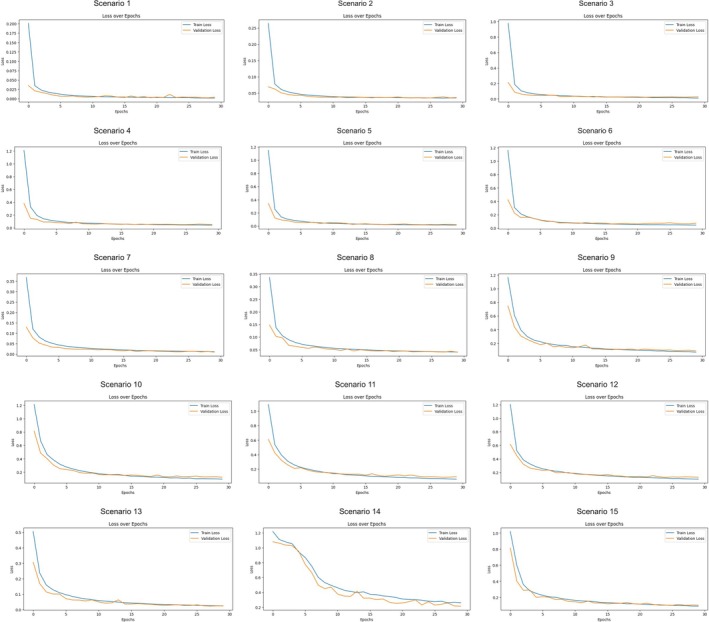
Training and validation loss curves across all experimental scenarios (1–15), illustrating model convergence behavior of the proposed HAT‐ECG framework.

**FIGURE 9:**
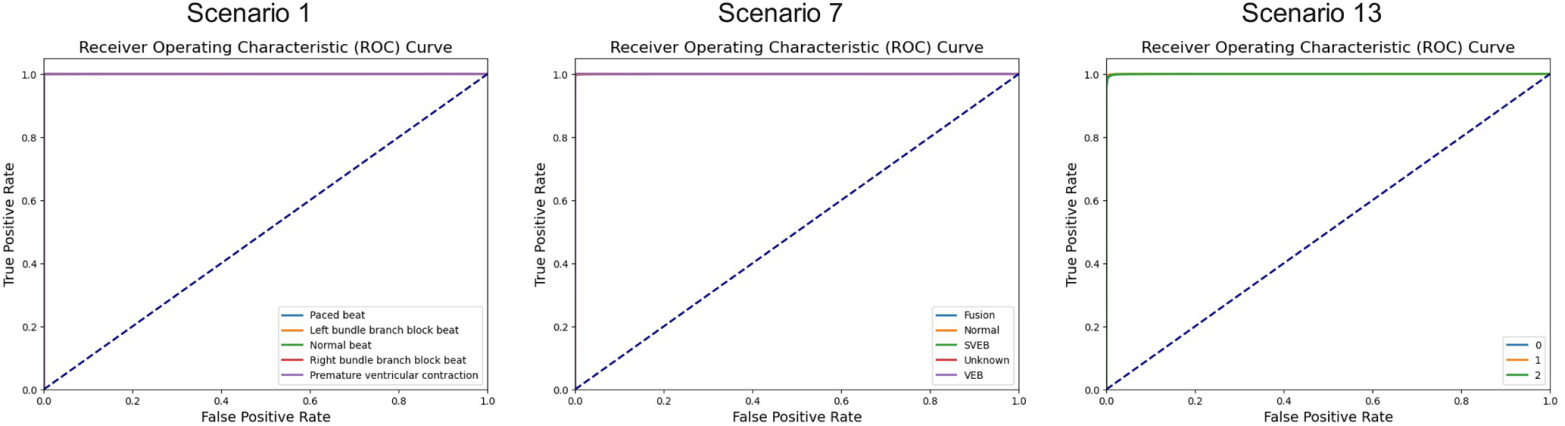
ROC curves for the best‐performing scenarios: Scenario 1—MIT‐BIH dataset (5‐class classification), Scenario 7—MIT‐BIH dataset (AAMI standard), and Scenario 13—INCART dataset (AAMI standard).

To analyze classification behavior, confusion matrices were generated for all experimental scenarios. Figure 6 shows results for MIT‐BIH under regular evaluation (Scenarios 1–6), AAMI standard evaluation (Scenarios 7–12), and INCART evaluation (Scenarios 13–15). These plots demonstrate consistent recognition across majority and minority arrhythmia classes, with minimal misclassification. Training and validation curves for accuracy and loss across all 15 scenarios are shown in Figures 7 and 8. Figure 9 presents the ROC curves for the best‐performing scenarios: Scenario 1 (MIT‐BIH, 5‐class classification), Scenario 7 (MIT‐BIH, AAMI standard), and Scenario 13 (INCART, AAMI standard).

### 3.7 Per‐class classification performance and beat‐type discrimination

To further investigate class‐wise discrimination behavior, a per‐class performance analysis was conducted using the confusion matrices of the best‐performing scenarios: Scenario 1 (MIT‐BIH, regular 5‐class classification), Scenario 7 (MIT‐BIH, AAMI‐standard classification), and Scenario 13 (INCART, AAMI‐standard classification). For each scenario, Precision, Recall, F1‐score, and Accuracy were computed individually for each beat category, enabling a detailed assessment of classification strengths and residual confusions.

As summarized in Tables 14, 15, 16, the proposed HAT‐ECG framework demonstrates consistently high discrimination capability across all beat types. In the MIT‐BIH regular evaluation (Scenario 1), Normal, LBBB, RBBB, PVC (VEB), and Paced beats all achieved Recall values above 97.9%, with particularly strong separation between ventricular ectopic beats and non‐ventricular classes. Under the AAMI‐standard evaluation (Scenario 7), the model effectively distinguishes SVEB and bundle branch block beats from VEB, achieving Recall and Precision values exceeding 99% for all major classes. Similarly, in the INCART evaluation (Scenario 13), the model maintained robust discrimination between Normal, SVEB, and VEB beats, confirming its generalization across datasets with higher physiological and acquisition variability.

**TABLE 14:** Per‐class performance metrics for Scenario 1 (MIT‐BIH dataset, regular 5‐class classification), derived from the confusion matrix of the proposed HAT‐ECG model.

Scenario	Metric	Class				
		N (Normal)	L (LBBB)	R (RBBB)	V (PVC)	/(paced)
1	Recall (%)	97.99	98.95	99.30	98.68	99.86
	Precision (%)	98.99	98.34	99.79	97.79	99.93
	F1‐score (%)	98.49	98.64	99.54	98.23	99.90
	Accuracy (%)	97.99	98.95	99.30	98.68	99.86

**TABLE 15:** Per‐class performance metrics for Scenario 7 (MIT‐BIH dataset, AAMI‐standard classification), highlighting discrimination among Normal, SVEB, VEB, Fusion, and Paced beats.

Scenario	Metric	Class				
		Fusion	Normal	SVEB	Unknown	VEB
7	Recall (%)	100.00	98.94	99.93	99.98	99.61
	Precision (%)	99.30	99.93	99.47	99.93	99.85
	F1‐score (%)	99.65	99.43	99.70	99.95	99.73
	Accuracy (%)	100	98.94	99.93	99.98	99.61

**TABLE 16:** Per‐class performance metrics for Scenario 13 (INCART dataset, AAMI‐standard 3‐class classification), demonstrating generalization across Normal, SVEB, and VEB beats.

Scenario	Metric	Class		
		N	S	V
13	Recall (%)	99.25	99.27	98.94
	Precision (%)	98.61	99.53	99.33
	F1‐score (%)	98.93	99.40	99.13
	Accuracy (%)	99.25	99.27	98.94

Notably, bundle branch block beats (LBBB and RBBB), which are mapped to the Normal superclass under the AAMI standard, were consistently distinguished from ventricular ectopic beats (VEB) with Recall values exceeding 99%, indicating that the proposed method does not conflate conduction abnormalities with ventricular arrhythmias.

## 4 DISCUSSION

The comparative analysis shows clear advantages of the proposed Hybrid Autoencoder‐Transformer (HAT‐ECG) framework over recent Transformer‐based ECG classification models. Across multiple configurations, the system achieved near‐perfect performance on the MIT‐BIH dataset, narrowing the gap to clinical‐grade detection thresholds. As shown in Table 17, HAT‐ECG reached an accuracy and F1‐score of 99.91%, outperforming prior state‐of‐the‐art models by a clear margin. Integrating unsupervised representation learning through the autoencoder with a compact Transformer classifier proved effective. The autoencoder compresses beat morphologies into discriminative latent representations, while the Transformer captures intra‐beat and inter‐beat dependencies through self‐attention mechanisms. This enables a deeper contextual understanding of temporal cardiac dynamics.

**TABLE 17:** Performance comparison on MIT‐BIH dataset (5‐class configuration).

Methods	Accuracy (%)	F1‐score (%)	ROC‐AUC (%)	Precision (%)	Recall (%)
Nikandish et al. (2024)	96.10	89.10	—	—	—
Hua et al. (2022)	99.24	99.00	—	—	99.00
Midani et al. (2023)	99.46	97.63	‐	98.26	97.01
**Proposed HAT‐ECG**	**99.91**	**99.91**	**100**	**99.91**	**99.91**

*Note*: The bolding indicates that these results are particularly noteworthy and represent key findings of our study.

A key insight is the operation of the Multi‐Head Attention mechanism, which dynamically focuses on distinct regions of the ECG signal and disregards irrelevant or noisy sections. Each attention head independently emphasizes specific morphological features, such as the P‐wave, QRS complex, or T‐wave, allowing the model to extract complementary diagnostic information. This adaptive attention enables automatic feature selection without manual region identification, which is important in ECG signals where clinically meaningful information is often localized within short, variable segments. As a result, HAT‐ECG can effectively distinguish among multiple arrhythmia types even when key features appear in different temporal locations.

Despite the strong empirical performance, the latent representations learned by the autoencoder are not explicitly constrained to correspond to predefined electrocardiographic descriptors, which limits direct physiological interpretability. In contrast to conventional 1‐D CNNs, whose localized convolutional filters inherently emphasize high‐amplitude, short‐duration components such as the QRS complex, the proposed autoencoder‐Transformer architecture is designed to integrate information across multiple temporal scales. The Multi‐Head Attention mechanism enables the model to attend dynamically to diverse waveform regions within each beat, allowing lower‐amplitude and longer‐duration components, including P‐wave and T‐wave morphology, to contribute alongside QRS‐related features. This architectural property provides a plausible explanation for the improved discrimination observed relative to CNN baselines, particularly for arrhythmias with heterogeneous or subtle morphological patterns. However, a quantitative decomposition of classification performance into contributions from specific ECG components or a direct mapping of latent dimensions to physiological markers would require dedicated explainable AI (XAI) analyses, such as attention attribution or waveform masking, which were beyond the scope of the present study.

Beyond feature extraction, the Multi‐Head Attention mechanism also aids adaptive denoising. By assigning lower attention weights to uninformative or noisy signal components, the model emphasizes salient morphological cues and reduces the influence of artifacts. This is especially valuable under the AAMI standard, where the number of arrhythmia classes increases and inter‐class feature overlap becomes more pronounced. As summarized in Table 18, the proposed method achieved 99.69% accuracy and F1‐score on the MIT‐BIH dataset under the AAMI five‐class configuration, outperforming previous works by more than 0.5% absolute accuracy. Focusing on discriminative subregions helps maintain high sensitivity and precision across diverse pathological patterns.

**TABLE 18:** Performance comparison on MIT‐BIH dataset using AAMI standard classification (5‐class configuration).

Methods	Accuracy (%)	F1‐score (%)	ROC‐AUC (%)	Precision (%)	Recall (%)
Zhang, Li, et al. (2025)	97.84	—	—	84.25	65.51
Shaker et al. (2020)	98.00	—	—	90.00	97.70
Sharma et al. (2021)	98.53	96.93	—	98.24	95.68
Najia & Faouzi (2025)	99.20	98.29	—	—	97.50
Yao et al. (2021)	99.61	99.42	—	—	99.33
**Proposed HAT‐ECG**	**99.69**	**99.69**	**100**	**99.69**	**99.69**

*Note*: The bolding indicates that these results are particularly noteworthy and represent key findings of our study.

The use of multiple attention heads promotes stability because each head captures distinct but complementary information. This reduces reliance on any single feature subset and improves robustness across patient populations and recording conditions. The model's consistent cross‐database performance reflects this property. When evaluated on the INCART database (Table 19), which includes greater channel diversity and signal variability, HAT‐ECG sustained 99.15% accuracy, demonstrating strong generalization across acquisition setups. The high Cohen's Kappa value further confirms the model's agreement with true clinical labels and emphasizes its reliability in multiclass classification tasks.

**TABLE 19:** Performance comparison on INCART dataset (AAMI standard and 3‐class configuration).

Methods	Accuracy (%)	F1‐score (%)	ROC‐AUC (%)	Precision (%)	Recall (%)
à Mougoufan et al. (2021)	95.43	—	—	—	—
Berrahou et al. (2024)	98.20	—	—	—	—
Mokhtari et al. (2025)	98.37	79.37	—	81.72	77.24
**Proposed HAT‐ECG**	**99.15**	**99.15**	**99.98**	**99.16**	**99.15**

*Note*: The bolding indicates that these results are particularly noteworthy and represent key findings of our study.

In addition to beat‐wise evaluation, a strict patient‐wise data partitioning strategy was employed to assess the true generalization capability of the proposed model in a clinically realistic setting. In this protocol, patients with comparable pathological conditions were distributed in a balanced manner across mutually exclusive training and test sets, ensuring that no subject contributed data to both phases. This design preserves the statistical distribution of arrhythmia patterns while enforcing evaluation on entirely unseen individuals during testing. Under the conventional random beat‐wise split, consistent with widely adopted evaluation protocols in previous studies on the MIT‐BIH dataset, the proposed model achieved an accuracy of 99.91%, reflecting strong performance under standard benchmarking conditions. However, beat‐wise evaluation may lead to optimistically biased performance estimates since beats from the same subject can simultaneously appear in both training and test sets, allowing partial subject‐specific information leakage. For this reason, beat‐wise evaluation is primarily used as a benchmarking framework to enable direct comparison with existing state‐of‐the‐art methods, rather than as a strict measure of clinical generalization. Beyond benchmarking, beat‐wise evaluation also has an important practical interpretation in patient‐specific cardiac monitoring applications. In particular, it is directly relevant to continuous arrhythmia surveillance systems based on portable and wearable ECG devices, such as Holter monitors (Pham et al., 2023), where beat‐by‐beat analysis is performed for a single individual over time. In such scenarios, a model trained on a broader patient population can be adapted for personalized monitoring, enabling highly sensitive detection of abnormal beats and timely clinical alert generation. This becomes especially valuable in hospital environments, where patients with a known cardiac history are continuously monitored using bedside or portable ECG systems, allowing clinicians to track cardiac activity with high temporal resolution and receive automatic alerts for potentially dangerous arrhythmic events. Moreover, given the low computational complexity of the proposed framework (0.021 GFLOPs), the method is suitable for deployment in resource‐constrained wearable and edge devices, where energy efficiency and real‐time inference are critical requirements. Together, these aspects indicate that while beat‐wise evaluation is methodologically important for benchmarking and system‐level deployment scenarios, it should be interpreted in conjunction with more stringent validation strategies to fully assess clinical reliability. In contrast, the patient‐wise evaluation represents a more stringent and clinically meaningful assessment of inter‐subject generalization. When applied, the proposed model achieved an accuracy of 90.81%, with an F1‐score of 92.61%, precision of 94.11%, and recall of 91.72%. This performance gap compared to beat‐wise evaluation reflects inter‐patient physiological variability rather than a model deficiency. In cardiac arrhythmia analysis, each patient exhibits a unique “cardiac signature” shaped by anatomical differences, clinical history, medication, and physiological state, resulting in subtle morphological variations of the same pathological condition across individuals. While beat‐wise evaluation may partially exploit patient‐specific characteristics, patient‐wise evaluation forces the model to rely exclusively on disease‐invariant representations. The observed performance under this setting demonstrates that the proposed framework successfully captures the shared pathological structure of arrhythmias while suppressing patient‐specific variability, which is a desirable property for real‐world clinical deployment.

While the proposed framework is not intended to provide new mechanistic insights into cardiac electrophysiology, its evaluation across heterogeneous datasets highlights its relevance to real‐world physiological variability. The strong generalization observed across MIT‐BIH, INCART, and PTB recordings suggests that the learned latent representations capture stable ECG morphological patterns that persist across patients, acquisition protocols, and noise conditions. Rather than explicitly modeling physiological processes, HAT‐ECG leverages data‐driven attention mechanisms to identify discriminative waveform characteristics that are clinically meaningful for arrhythmia classification. This methodological focus aligns with the intended scope of the study and supports potential use in automated screening or decision‐support settings. At the same time, reliance on publicly available datasets represents a limitation, and future work should include validation on more recent and longitudinal ECG cohorts to further assess patient‐specific and temporal physiological variations.

We further analyzed per‐class performance against a representative 1‐D CNN baseline. Hua et al. (2022) report a 1‐D CNN evaluated on the same MIT‐BIH dataset using the identical five beat classes (Normal, LBBB, RBBB, PVC, and Paced). Using the published confusion matrix, we recomputed per‐class recall, precision, F1‐score, and accuracy, and compared them with corresponding metrics obtained from HAT‐ECG. As summarized in Table 20, the proposed method consistently outperformed the 1‐D CNN across all beat categories. The most pronounced improvements were observed for PVC and LBBB beats, which are known to exhibit higher intra‐class variability and morphological complexity. These results suggest that the autoencoder‐transformer architecture is particularly effective at modeling subtle and heterogeneous ECG patterns that are more challenging for purely convolutional models.

**TABLE 20:** Per‐class performance comparison between a 1‐D CNN baseline (Hua et al., 2022) and the proposed HAT‐ECG model on the MIT‐BIH five‐class beat classification task.

Method	Metric	Class				
		N (Normal)	L (LBBB)	R (RBBB)	V (PVC)	/ (paced)
1D CNN Hua et al. (2022)	Recall (%)	97.99	98.95	99.29	98.68	99.86
	Precision (%)	98.99	98.34	99.79	97.79	99.93
	F1‐score (%)	98.49	98.64	99.54	98.23	99.90
	Accuracy (%)	99.39	99.49	99.30	99.30	99.86
Proposed Method	Recall (%)	99.64	99.98	100.00	99.91	100.00
	Precision (%)	100.00	99.91	99.96	99.75	99.97
	F1‐score (%)	99.81	99.95	99.98	99.83	99.98
	Accuracy (%)	99.91	99.97	99.98	99.97	99.98
Amount of Improvement	Recall (%)	+1.65	+1.03	+0.70	+1.23	+0.14
	Precision (%)	+1.01	+1.57	+0.17	+1.96	+0.04
	F1‐score (%)	+1.32	+1.31	+0.44	+1.60	+0.08
	Accuracy (%)	+0.52	+0.48	+0.70	+0.67	+0.14

Additionally, Table 21 showcases the performance comparison on the PTB dataset (2‐class configuration). The proposed HAT‐ECG architecture achieved an Accuracy of 98.45%, Recall of 97.33%, Precision of 99.56%, and an ROC‐AUC of 99.85%. Another notable finding is the model's resilience to variable‐length segmentation. Although fixed 300‐sample windows yielded the best performance, variable‐length segments (300–400 samples) slightly reduced accuracy but substantially improved generalization. This suggests the network learned invariant morphological representations resilient to moderate temporal shifts, which is essential for wearable and ambulatory ECG monitoring where beat durations fluctuate due to physiological and motion‐related factors.

**TABLE 21:** Performance comparison on PTB dataset (2‐class configuration).

Methods	Accuracy (%)	F1‐score (%)	ROC‐AUC (%)	Precision (%)	Recall (%)
Sharma et al. Sharma et al. (2021)	88.59	88.69	—	87.34	90.07
Rafi et al. Rafi & Ko (2022)	98.15	97.79	—	97.31	96.85
Pham et al. Pham et al. (2023)	98.28	—	—	99.90	97.72
**Proposed HAT‐ECG**	**98.45**	**98.44**	**99.85**	**99.56**	**97.33**

*Note*: The bolding indicates that these results are particularly noteworthy and represent key findings of our study.

The low computational cost (0.021 GFLOPs in total) shows that HAT‐ECG achieves a strong balance between accuracy and efficiency. Compared with conventional deep neural networks that rely on heavy convolutional layers, this architecture reduces complexity while preserving diagnostic precision. This efficiency enables real‐time inference on resource‐constrained platforms, making HAT‐ECG suitable for edge deployment and continuous monitoring in wearable healthcare systems.

It is important to contextualize the proposed HAT‐ECG framework with respect to commercial beat‐to‐beat discrimination algorithms currently deployed in Holter monitoring systems (Huang et al., 2024). Existing commercial solutions are typically engineered for robustness, interpretability, and regulatory compliance, often relying on a combination of rule‐based heuristics, handcrafted morphological features, and conservatively tuned machine learning models that have been validated through extensive clinical testing. These systems are optimized for stable long‐term monitoring in real‐world clinical environments rather than for maximizing classification performance on benchmark datasets.

In contrast, HAT‐ECG is a research‐oriented framework designed to investigate how modern deep representation learning and attention‐based architectures can enhance arrhythmia discrimination under challenging conditions, such as high inter‐patient variability, subtle morphological differences, and class imbalance. The proposed model is not intended to replace existing commercial Holter algorithms, but rather to complement them by providing insights into data‐driven feature learning and by serving as a potential upstream analytical module for candidate beat screening, decision support, or offline analysis. The lightweight computational footprint of HAT‐ECG suggests feasibility for edge deployment; however, translation into commercial systems would require prospective clinical validation, explainability assessments, and regulatory approval. As such, the primary contribution of this work lies in advancing methodological understanding and performance potential, rather than proposing immediate replacement of established clinical technologies.

In summary, by combining variable‐length beat‐centered segmentation, autoencoder‐based latent feature learning, and Transformer‐driven temporal modeling, HAT‐ECG advances both the accuracy and practical deployability of automated arrhythmia detection. The synergy between unsupervised representation learning and attention‐based feature selection yields a compact yet powerful model capable of clinical‐grade classification.

## 5 CONCLUSION

This study introduced HAT‐ECG, a lightweight Hybrid Autoencoder‐Transformer framework for ECG arrhythmia classification. By combining unsupervised latent feature learning with Multi‐Head Attention–based temporal modeling, the proposed approach effectively captures both local waveform morphology and long‐range cardiac dependencies. Experimental evaluations across multiple benchmark ECG datasets demonstrated strong classification performance and robust generalization under different evaluation settings. The attention mechanism enables adaptive focusing on diagnostically relevant ECG regions, improving discrimination among arrhythmia types while maintaining resilience to signal variability and noise. These findings suggest that the learned representations preserve clinically meaningful cardiac patterns relevant to automated arrhythmia analysis. Owing to its low computational complexity and stable performance, HAT‐ECG shows potential for real‐time intelligent cardiac monitoring and wearable healthcare applications. Future work will focus on explainable AI analysis and validation on larger fully patient‐independent clinical cohorts and scenarios.

## AUTHOR CONTRIBUTIONS

**Shahin Sharbaf Movassaghpour:** Data curation; methodology; resources; software. **Masoud Kargar:** Conceptualization; formal analysis; investigation; methodology; project administration; supervision; validation; visualization. **Ali Bayani:** Visualization.

## FUNDING INFORMATION

This research was conducted without external funding or institutional support.

## CONFLICT OF INTEREST STATEMENT

The authors declare that they have no conflicts of interest to disclose.

## ETHICS STATEMENT

This research utilizes the publicly available MIT‐BIH Arrhythmia, St Petersburg INCART 12‐lead Arrhythmia, and PTB Diagnostic ECG datasets for ECG signal analysis in cardiac disease diagnosis. The study adheres to relevant regulations and the ethical guidelines of Physiological Reports. No direct human interactions were involved.

## Data Availability

The MIT‐BIH Arrhythmia dataset used in this research is available at the following DOI: https://doi.org/10.13026/C2F305. St Petersburg INCART 12‐lead Arrhythmia dataset is accessible via the following DOI: https://doi.org/10.13026/C2V88N. PTB dataset is accessible via the following DOI: https://doi.org/10.13026/C28C71. The source code and models developed in this research are available at https://github.com/MasoudKargar/HAT‐ECG.
